# Effects of a Multicomponent Exercise Program on Improving Frailty in Post-COVID-19 Older Adults after Intensive Care Units: A Single-Group Retrospective Cohort Study

**DOI:** 10.3390/biology11071084

**Published:** 2022-07-20

**Authors:** Juan Nicolás Cuenca-Zaldivar, Álvaro Monroy Acevedo, Josué Fernández-Carnero, Eleuterio A. Sánchez-Romero, Jorge Hugo Villafañe, Carlos Barragán Carballar

**Affiliations:** 1 Research Group in Nursing and Health Care, Puerta de Hierro Health Research Institute—Segovia de Arana (IDIPHISA), 28222 Majadahonda, Spain; jcuenzal@yahoo.es (J.N.C.-Z.); alvaromonroy1@gmail.com (Á.M.A.); 2 Rehabilitation Service, Guadarrama Hospital, 28440 Madrid, Spain; 3 Department of Physical Therapy, Occupational Therapy, Rehabilitation and Physical Medicine, Universidad Rey Juan Carlos, 28922 Alcorcón, Spain; 4 Department of Physiotherapy, Faculty of Sport Sciences, Universidad Europea de Madrid, 28670 Villaviciosa de Odón, Spain; carlos.barragan.carballar@gmail.com; 5 Musculoskeletal Pain and Motor Control Research Group, Faculty of Sport Sciences, Universidad Europea de Madrid, 28670 Villaviciosa de Odón, Spain; 6 Department of Physiotherapy, Faculty of Health Sciences, Universidad Europea de Canarias, 38300 Santa Cruz de Tenerife, Spain; 7 Musculoskeletal Pain and Motor Control Research Group, Faculty of Health Sciences, Universidad Europea de Canarias, 38300 Santa Cruz de Tenerife, Spain; 8 IRCCS Fondazione Don Carlo Gnocchi, Piazzale Morandi 6, 20148 Milan, Italy; mail@villafane.it; 9 Escuela Internacional de Doctorado, Department of Physical Therapy, Occupational Therapy, Rehabilitation and Physical Medicine, Universidad Rey Juan Carlos, 28922 Alcorcón, Spain; 10 OnelifeCenter, Multidisciplinary Center for the Prevention and Treatment of Pain, 28924 Alcorcón, Spain

**Keywords:** COVID-19, frailty, exercise, physical activities

## Abstract

**Simple Summary:**

Older adult patients with post-COVID-19 syndrome present greater physical impairment accompanied by frailty, which is why multicomponent exercise programs (MEP) are recommended for their positive effects on improving frailty and physical capacity. The aim of this study was to evaluate the effects of a short MEP (Vivifrail; <4 weeks) on the improvement of physical performance and frailty in post-COVID-19 older adults in a hospital setting. Data were collected from the functional gait training program based on selected Vivifrail MEP in a single group, applied to patients admitted with a diagnosis of post-COVID-19 functional impairment. The program was carried out for 3 weeks, with daily sessions lasting 40 min. Patients included were assessed at the beginning and at the end of the protocol on their functionality, strength, balance, gait and coordination, and ability to carry out basic activities of daily living. The results of this study show significant improvements in physical fitness and frailty by means of the MEP applied in its short version (<4 weeks) showing even clinically relevant improvements. The MEP is effective and safe on improving frailty in post-COVID-19 older adult patients.

**Abstract:**

Background: Older adult patients with post-COVID-19 syndrome present greater physical impairment accompanied by frailty than younger patients, which is why multicomponent exercise programs (MEP) are recommended for their positive effects on improving frailty and physical capacity. The aim of this study was to evaluate the effects of a short MEP (Vivifrail; <4 weeks) on improving frailty in post-COVID-19 older adults after intensive care units. Methods: To develop a retrospective cohort study, data were collected from the functional gait training program based on selected Vivifrail MEP in a single-group and applied to patients admitted with a diagnosis of post-COVID-19 functional impairment. The MEP was carried out for 3 weeks, with daily sessions lasting 40 min. Patients included were assessed at the beginning and at the end of the protocol by using the Short Performance Physical Battery (SPPB), the number of falls in the last year, the number of falls with medical attention, the Timed Up and Go (TUG) test, the presence of dementia, the Trunk Control Test (TCT), the Tinetti balance and gait test, Barthel Index, Medical Research Council Sum Score (MRCSS) and handgrip strength dynamometry. Results: The results of this study show statistically significant improvements in physical fitness and frailty with increases in the Short Physical Performance Battery (Z = 9.12, *p* < 0.001) by means of the MET applied in its short version (<4 weeks) showing even clinically relevant improvements (>2.5 points). Statistically significant improvements were also found in Medical Research Council Sum Score (Z = 12.345, *p* < 0.001), Barthel Index Score (Z = 12.272, *p* < 0.001), Trunk Control Test (Z = 12. 36, *p* < 0.001), Tinetti–POMA (Z = 12.293, *p* < 0.001) including the balance (Z = 12.11, *p* < 0.001), gait (Z = 12.164, *p* < 0.001) subscales and in the hand dynamometry (Z = 12.172, *p* < 0.001). Conclusions: The selected Vivifrail MEP is effective and safe for improving frailty in post-COVID-19 older adult’s patients.

## 1. Introduction

SARS-CoV-2, also known as COVID-19, causes severe acute respiratory syndrome that most often affects patients over 60 years of age and those with underlying medical conditions. The elderly are the most vulnerable to the negative consequences of severe acute respiratory syndrome due to SARS-CoV-2 infection [1]. In an observational study in Spain that determined intra-hospital mortality related to COVID-19 in older people, researchers found that functional impairment as measured by the Barthel Index or the presence of frailty are predictive factors influencing higher mortality [2]. Others have shown, in a multicenter European study conducted at 11 hospitals in the UK and Italy, that patients with COVID-19 classified as frail are more likely to die and the time to discharge from the hospital is less rapid than those who are not frail [3]. In this study, a sample of 1564 patients with COVID-19 showed that the prevalence of frailty is 49.4% and that frailty was associated with earlier death.

Clinical symptoms associated with COVD-19 mainly affect the respiratory track, but symptoms are highly heterogeneous, from no symptoms to minimal symptoms, to mild symptoms, to severe symptom such as pneumonia, acute respiratory distress syndrome, septic shock, and, potential systemic multiple organ failure syndrome [4] A recent study has found that COVID-19 can accelerate the deterioration of physical performance and frailty in older people in nursing homes by about 20%, a 22% decrease in walking speed and a 21% increase in frailty compared to healthy older people of the same age [5]. As a result of immobilization and prolonged mechanical ventilation (MV), the recovery of respiratory and physical functions may take a long time after patient discharge from intensive care unit (ICU), or sometimes only a partial recovery is achieved, leading to a reduction in quality of life [6].

Both the lack of mobility associated with confinement and bed immobilization linked to longer hospital admissions have been shown to have a direct effect on muscle mass and physical function, even in young adults [7]. It has been shown that the loss of muscle mass is more accentuated in the first 2–3 weeks of immobilization and that young adults recover better from the sequelae but not all recover initial levels [7,8]. Patients admitted to the ICU have their frailty condition accelerated, with 8% dying at 2-year follow-up and 19% at 5 years [8].

Guidelines have been published for the approach to patients with COVID-19 wherein exercise is included as one of the fundamental pillars, in addition to interventions in respiratory physiotherapy [9]. In this sense, international multicentre observational cohort studies have been launched to analyse the effects of rehabilitation treatment, including physiotherapy based on physical exercise and occupational therapy among others, on patients’ recovery in activities of daily living [10]. The Vivifrail program is a multicomponent exercise program (MEP) whose main objective is to improve the health of older adults by preventing and treating frailty and the risk of falls. This program combines strength, endurance, balance and gait training, producing significant improvements in functional capacity [11]. This program has been conducted in hospitalized older adult patients with favorable results in functional capacity, frailty, muscle strength, cognitive status, gait, and balance. In a recent study of older people with sarcopenia, they were given two different forms of the MEP exercise program (4 weeks vs. 24 weeks). They found positive results with both doses of the Vivifrail MEP, reviving frailty in 36% of cases and 59% improved until reaching full autonomy [12]. However, no studies have been conducted to analyze the effectiveness of this program in frail older adult survivors of SARS-CoV-2 [11]. On the other hand, the effect of physical activity on pain is known, but this approach has not been performed in SARS-CoV-2 survivors [13]. Although there are not many published trials on exercise in post-COVID-19 patients, clinical guidelines and recommendations have been published indicating the use of exercise for these patients [14].

Therefore, the aim of the present study was to assess the effects of a MEP (Vivifrail) short-programme (<4 weeks) on the improvement of frailty in post-COVID-19 older adults after intensive care units.

## 2. Materials and Methods

### 2.1. Study Design

A single-group, retrospective cohort study was developed following Strengthening the Reporting of Observational Studies in Epidemiology Guidelines, (STROBE) [15]. For the review of clinical histories and data exploitation, we had the approval of the Guadarrama Hospital Center Management and with the approval of the Research Ethics Committee of the Hospital Universitario Puerta de Hierro de Majadahonda, Madrid. (24 November 2021). At all times the confidentiality of the information was preserved, making responsible use of the data, as established by current Spanish regulations and in accordance with the Declaration of Helsinki.

### 2.2. Study Population

Data were collected from the functional gait training programme based on Vivifrail MEP carried out at Guadarrama Hospital as part of routine physiotherapy clinical practice, applied to older adult patients admitted with a diagnosis of post-COVID-19 functional impairment to Guadarrama Hospital between July and October 2020.

The inclusion criteria required patients older than 50, admitted to the Hospital de Guadarrama after discharge from intensive care units (ICUs) for rehabilitation who presented a diagnosis of post-COVID-19 functional impairment. For exclusion criteria, the following were not included: (a) patients with acute locomotion disorders with contraindications for lower limb exercise; (b) patients unable to walk; (c) patients with severe pain in the lower limb (score above 7 on the 10-point visual analog scale); and (d) patients with moderate or severe cognitive impairment that altered comprehension and collaboration in the implementation of the exercise program (Mini Mental Status Examination score less than 24 points).

### 2.3. Outcomes Measures

Patients included were assessed at the beginning and at the end of the protocol by using the following scales.

### 2.4. Physical Functioning

The primary outcome through the Short Performance Physical Battery (SPPB) [16], composed of a series of assessments of lower extremity functioning in older adults that evaluated the transition from sitting to standing position with the feet in three different positions (side-by-side or Romberg test, tandem, and semi-tandem), the time to walk 4 m (4-m gait speed test), and to stand up from a chair five times (chair stand-up test) by using a maximum score of 12, with higher scores indicating high functionality in the lower limb.

The following secondary outcomes were also collected.

### 2.5. Risk of Falls

Assessment of fall risk according to the Vivifrail protocol: cumulative number of falls in the past year, cumulative number of falls with physician care, suffer at least 1 fall in the past year, Timed Up and Go test (TUG) [17] and existence of dementia to establish the existence of fall risk (+ or −); the TUG assesses the time it takes to get out of the chair, walk three meters, back to the chair, and to sit down.

### 2.6. Balance

The Trunk Control Test (TCT) [18] evaluated four aspects of trunk movement, swinging to both sides, sitting balance, and rising from the floor; individual items are scored from 0 (incapable) to 12 (ability to perform the movement but with an unusual style) and 25 (ability to perform the movement correctly).

The Tinetti balance and gait test evaluated the gait and balance abilities of the older adult; it is composed of 16 items, 9 for balance and 7 for gait, each of which is scored on a 3-point ordinal scale from 0 to 2, where the higher score denotes independence on each item [19].

### 2.7. Basic Activities in Daily Life

The Barthel Index evaluated one’s ability to care for him/herself through ten activities of daily living like feeding, bathing, grooming, dressing, bowel and bladder control, toileting, chair transfer, ambulation, and stair climbing; the maximum score is 100 points and the higher the score, the greater the functional independence of the patient. The following baseline data regarding age, sex, and admission diagnosis were also collected [20].

### 2.8. Strength

The Medical Research Council Sum Score (MRCSS) strength assessment scale evaluated muscle strength in the upper and lower limbs. The different movements evaluated are scored from 0 to 5 and a maximum score of 60 can be obtained [21].

Handgrip strength was used by averaging the result of three attempts with the dominant hand by using a Baseline© BIMS Digital 5-Position Grip Dynamometer, Clinic Model, 1072 Tafelski Rd New Waverly, TX, 77358-4500 United States [22,23].

### 2.9. Multicomponent Physical Exercise Intervention

The patients included received an individualised work programme, selected from the A+ and B+ levels of the Vivifrail MEP. The Vivifrail MEP levels define the patient’s level of independence. Level A indicates that the patient has difficulty standing, level B indicates that the patient walks with diffimathvariant="bold-script"ulty or with aids, level C represents those patients with mild difficulty walking or standing, and level D indicates that physical limitations are minimal or non-existent. The + symbol also indicates that there is a risk of falling.

The exercises were selected by a consensus panel formed by professionals from the rehabilitation service of the Guadarrama Hospital when the Vivifrail program was implemented in the hospital in 2017. The selection was based on an evaluation of the most frequent levels of use of the Vivifrail MEP and the exercises considered easiest both in terms of physical execution and comprehension were selected. This protocol was applied with good results in patients with hip fracture and is the one used as usual in the physiotherapy service of the Guadarrama Hospital [24]

The MEP was carried out for 3 weeks (15 days), from Monday to Friday, with sessions lasting 40 min. In weeks 1 and 2, the exercises are those of an A+ patient and in week 3 the level of difficulty is increased with exercises for B+ patients.

Patients included in the program received the following physiotherapy treatment.

**Week 1:**
**1. Strength exercises:** The patient performs seated exercise of flexion and extension of the arms with an elastic band in 2 series of 10 repetitions, holding for 5 s **2. Seated exercise:** A diagonal opening of the arms by using an elastic band series of 10 repetitions, holding for 5 s. **3. Cardiovascular exercises:** The cardiovascular exercise program should only be started when the elderly person has improved muscle strength, walking for 15 s and resting for 10 s, repeating 7 times. **4. Balance and walking:** The patient stands near a table, a wall or a family member, placing the heel of one foot in contact with the toe of the other foot. This involves taking small steps in a straight line, placing the heel of the advancing foot just in front of the toe of the other foot. If the patient progresses in safety, it is placed on a table or railing, maintaining 10 s with each foot and repeats 3 times.
**Week 2:**

The same exercises as in week 1 were repeated by all patients, with increasing load.

**1. Strength exercises:** Three sets of ten repetitions of all exercises, holding for 5 s. **2. Cardiovascular exercises:** Walking 30 s and resting 20 s, repeating 5 times. **3. Balance and gait exercises:** Holding 20 s with each foot, repeating 3 times.
**Week 3:**
**1. Strength exercises:** Leg bending exercise without a chair standing behind a table. The patient begins by flexing the hips and knees as if he were going to sit down, then returns to the initial position, and if necessary, is helped by a chair behind him for safety, for 2 sets of 10 repetitions. In addition, the patient goes up and down stairs for 11 steps, repeating 2 times. **2. Cardiovascular exercises:** The patient walks for 30 s and rests for 20 s, repeating 5 times. **3. Balance and walking exercises:** The patient maintains balance on one leg and with arms crossed, holds for 10 s with each leg, repeating 2 times.

### 2.10. Statistical Analysis

For the statistical analysis, the R Ver. 3.5.1. (R Foundation for Statistical Computing, Institute for Statistics and Mathematics, Welthandelsplatz 1, 1020 Vienna, Austria) was used. The level of significance was established at *p* < 0.05. The distribution of quantitative variables, both baseline and outcome, was tested with the Kolmogorov–Smirnov test with Lilliefors correction which showed the non-normal distribution of all the result variables. The qualitative variables were described in absolute values and frequencies and the quantitative variables with mean and standard deviation. For the pre-post-intervention qualitative variables, a nominal symmetry test was used for related data, equivalent to the McNemar and McNemar–Bowker tests, due to the presence of asymmetric contingency tables or with cells with values equal to 0. In the variables with more than two categories, the corresponding post hoc tests with Bonferroni correction were performed. The Wilcoxon Signed-Rank test was applied to quantitative variables. The effect size in the quantitative variables was defined with the r statistic, as 0.1–0.4 (small), 0.4–0.6 (moderate), and >0.6 (large). The strength of association in the categorical variables was defined with Cohen’s G as <0.15 (small), 0.15–0.25 (moderate), and >0.25 (large). Likewise, the risk of falls was analyzed by using the cut-off points proposed for the total score of the Tinetti test of balance and gait and in the TUG test [25].

## 3. Results

### Patient Flow and Principal Characteristics

A total of 101 patients participated with the eligibility criteria and were included in the MEP, balanced between 56 men (55.4%) and 45 women (44.6%) with an age of 73.09 ± 12.62 (54–93) years and a body mass index of 25.44 ± 4.05. The duration of the ICU stay was 19.74 ± 29.42 days. The main reason for admission was physical deterioration after pneumonia (49.5%) associated with a stay in the ICU (44.6%) and polyneuropathy of the critical patient (16.8%), with a history of arterial hypertension (61.4%), dyslipidemia (35.6%), diabetes mellitus (33.7%), obesity (18.8%), and heart failure (13.9%) (Table 1).

Significant changes occurred pre-post-treatment in the quantitative outcome variables, with significant moderate to large effect sizes. There were significant increases in the Short Physical Performance Battery (Z = 9.12, *p* < 0.001) with improvements in all items, Medical Research Council Sum Score (Z = 12.345, *p* < 0.001), Barthel Index Score (Z = 12.272, *p* < 0.001), Trunk Control Test (Z = 12.36, *p* < 0.001), Tinetti-POMA (Z = 12.293, *p* < 0.001) including the balance (Z = 12.11, *p* < 0.001), gait subscales (Z = 12.164, *p* < 0.001), and in the hand dynamometry (Z = 12.172, *p* < 0.001) (Table 2).

In the qualitative outcome variables, there were significant pre-post treatment changes in all except the Categorized Timed Up and Go test with large effect sizes. In the Short Physical Performance Battery items there were increases in the proportion of patients with long times in the bipedal position (Categorized Romberg’s test, Categorized Tandem test and Categorized Semitandem Test more than 9–10 s) and in the proportion of patients with lower times in the dynamic tests of the same (Categorized 4-Meter Walk Gait less than 4.82 s and Categorized Chair Stand test 11.20–16.69 s). There was also a decrease in the proportion of patients with a Vivifrail level A+ and a significant increase in those with a C and D levels. In the Categorized Barthel Index score, the proportion of patients with moderate to slight dependence or independent increased and the number of those who presented severe dependence or total dependence decreased (Table 3).

Regarding the risk of falls, there were significant changes pre-post-treatment in the risk level both measured with the Timed Up and Go test and with the Tinetti-POMA, increasing the proportion of patients with low to moderate risk or without risk and decreasing the most dependent or with high risk (Table 4).

**Figure 1:**
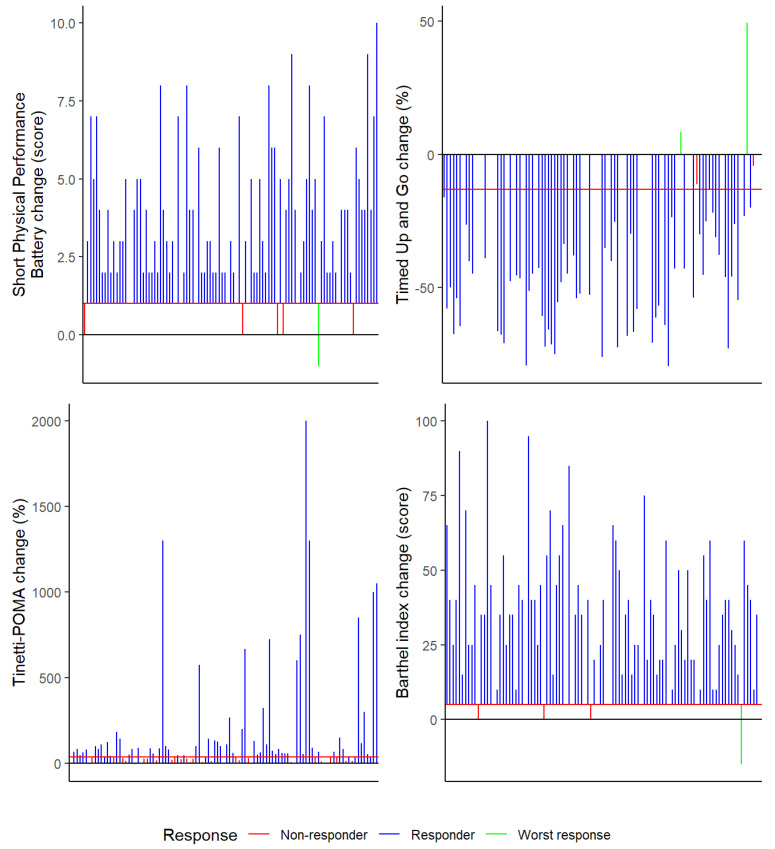
Responders and non-responders rate.

The responders rate is clearly higher than that of non-responders in all tests and only a small number of worst responses at the end of the study are observed, less than 2% in variables, 2 (1.98%) patients in Tinetti–POMA and Timed Up and Go and 1 (0.99%) patient in SPPB and Barthel Index respectively (Figure 1).

Given the small number of patients affected and the heterogeneity of the 4 scales (SPPB, TUG, Tinetti–POMA and Barthel Index), we did not explore whether there were any common clinical features. None of them had myasthenia gravis, and this worse response indicates that in some scales these patients achieved worse scores compared to baseline.

## 4. Discussion

The results of the present study show the efficacy of the MEP at the pre-post assessment based on Vivifrail MEP on improving frailty in post-COVID-19 older adults after intensive care units. Moreover, less than 2% of patients present a worst response at the end of program and none of them discontinued it due to any adverse event, which gives the MEP the necessary safety to be applied in post-COVID-19 older adult patients, in addition to its low cost. To our knowledge, this is the first time that an analysis of the adverse effects of a MEP based on the Vivifrail MEP protocol has been performed, and research into its efficacy and safety in post-COVID 19 older adults should be further developed.

The results of the present study especially reflect a significant improvement in the functional ability to maintain balance and gait.

In relation to the functional variable (SPPB), a previous study developed by Udina et al. [26] applied a MEP based on strengthening, aerobic exercise, and balance for 10 days and found a clinically significant improvement of 2.5 points in the post-COVID-19 patients who did not come from an ICU and 3.7 points in the group of patients derived from an ICU. In our study, we found a similar improvement, also increasing by 2.5 points on average in this series of assessments of lower extremity function, although we applied 3 weeks of a MEP as opposed to only 10 days in the Udina et al. study. A previous study by Martínez–Velilla et al. [27] developed in 370 older adult patients hospitalised at acute care units have found similar results with MEP on the SPPB scale and on the Barthel Index in elderly patients but without post-COVID 19 syndrome, with respect to the usual care group. In a recent Delphi Expert Consensus and in a recent scoping review, developed rehabilitation recommendations that recommend exercise as one of the therapies to be used in post-COVID-19 patients [15,28] are discussed.

In the variable of basic activities of daily living we found an improvement of 33 points, and these data are higher than those found in the previous study by Udina et al., who found an improvement of 18 points [26]. These authors conducted a study on the treatment of 33 adult post-COVID-19 patients by using a protocol very similar to the present study, together with combined resistance and endurance and balance training, but applied 7 days a week, for an average of 8.2 ± 1.7 days. This difference may be due to the fact that MEP lasted 3 weeks in the present study, twice as long as in the Udina et al. study, and the more medium-term effects of MEP may have influenced this.

The Vivifrail MEP has demonstrated short-term efficacy (4 weeks) in sarcopenic, frail, and institutionalized older adults, with health improvements being maintained after 14 weeks of inactivity due to COVID-19 confinement, and may have prevented severe functional decline and loss of strength [29]. Similar to the present study, the Vivifrail MEP has demonstrated efficacy as a treatment for dinapenia and dependence in ambulatory older women, significantly improving their functionality in muscle strength, gait speed (3 mt and 6 mt), Chair Stand, and TUG tests [30]. In addition, the Vivifrail MEP has demonstrated efficacy and safety in improving measures of physical/cognitive functioning and quality of life in older adults with non-small-cell lung cancer as an adjuvant or palliative treatment [31]. Apostolo et al. [32] demonstrate clear evidence for the utility of exercise therapy interventions to treat frailty, also from an economic perspective. Compared to usual care, Apostolo’s interventions provided better value for the money, and had favorable effects on some of the frailty-related outcomes in inpatient and outpatient management without increasing costs, supporting the clinical investment of resources in frailty interventions.

In relation to walking speed measured with the TUG test, we found an improvement of 8 s on average at the end of treatment with the MEP. Although there are no previous studies in patients with COVID-19 with which we can compare this parameter of functionality, in a study by Romero–García et al. [29] in which they applied similar MEP but in elderly people with dynapemia, they found an improvement in the TUG test of 2 s on average. However, their patients started the treatment with a walking speed of 10.14 s on average, which is much lower than the speed they had in our sample of 23.43 s at baseline. This was more than in our study, which only lasted 3 weeks, as compared to Romero–Garcia et al. who did 12 weeks.

Regarding the most effective dose of MEP to curb the effect of hospitalization in older adults, Echeverria et al. [33] determined that after 6 weeks of intervention through MEP, functional and nutritional benefits similar to those obtained in a group of older adults who performed the same exercise protocol for 12 weeks were generated. In the present study, the MEP lasted 3 weeks, sufficient time to obtain statistically significant improvements and effect sizes in the physical variables measured, which invites future research to find the appropriate dose in older adult post-COVID-19.

Courel–Ibañez et al. [12] suggest that to prevent our older adults from a significant functional decline, an intervention of at least 4 weeks, 3 times a year (avoiding more than 14 weeks of inactivity between periods) will be determinant.

Sáez de Asteasu et al. [34] examined individual response by comparing usual care with a Vivifrail MEP programme in older adult patients hospitalized in the acute unit, presenting a higher prevalence of responders and a lower prevalence of non-responders and worst responders for functional capacity, muscle strength, and cognitive function in the Vivifrail MEP group. Of great importance is the association that this group of researchers found between presenting a worst response in functional capacity to physical exercise or usual care during hospitalization and mortality at 1 year after discharge. In the present study, we found only 1–2% of worst responders, 3–34% of non-responders, and 65–95% of responders for the variables Short Physical Performance Battery score, TUG and Tinetti tests, and Barthel Index score. According to other studies, these results suggest not only an improvement in frailty, but also a prevention of mortality in almost all of the post-COVID-19 older adult patients included in the present study [35,36,37].

It appears that exercise therapy is effective as a treatment in post-COVID-19 patients, as demonstrated in studies such as that of Daynes et al. [38], using a two session per week, six-week rehabilitation program of aerobic exercise (walking), and upper and lower limb strength training found that improvements in exercise capacity and symptoms of dyspnea, fatigue, and cognition in post-COVID-19 patients. However, in the present study, it can be observed that by introducing a MEP through a three-week intervention, which is based on the training of different parameters and physical qualities through strength exercises, cardiovascular exercises, balance and gait, improvements are achieved that really make an impact on fragility by improving functional capacity, balance, and ability in basic activities of daily living.

The lack of information related to all fields of post-COVID-19 infection rehabilitation creates the need for a broad collaborative network to provide urgent answers related to this disease.

Although the benefits of regular therapeutic exercise as a multisystem anti-aging agent are already known, more research is needed to understand and implement effective therapeutic exercise interventions for post-COVID-19 older adults [39].

Furthermore, considering that global healthcare spending is expected to continue to grow, but that the available budget in countries is uneven, the development of economical and effective therapies such as the MEP is well suited to address many of the post-COVID-19 neuromusculoskeletal sequelae in the case of patients living in more vulnerable countries [40].

### 4.1. Limitations

A major limitation was that the sample size of post-COVID-19 older adults in the study was small and heterogeneous, given that a clinical study of 101 cases was presented. Another important limitation is that no control or placebo group was included to compare with the natural evolution or with other interventions as well as to know the specific effect of this MEP.

Finally, although there is an inherent bias in the quality of information that exists in any retrospective study design, this bias has been minimized in the present study by using a very complete and reliable data source.

### 4.2. Clinical Implications

According to a recent systematic review and meta-analysis, these results can clarify that low load exercises have been shown to produce considerable improvements in strength [41]. This is important because the exercises included are of low intensity and, on the one hand, that people with post-COVID-19 syndrome are more reluctant to exercise due to fatigue and low exercise intolerance. These programs can be an interesting way for these patients to obtain functional improvements. On the other hand, the implementation of exercise programs has been shown to reduce the risk of acquiring infectious diseases and even mortality from infectious diseases and therefore may act as a preventive method to reduce the risk of re-infection [42].

## 5. Conclusions

Supervised and personalized MEP (Vivifrail) in older adult survivors of COVID-19 showed positive effects in a pre-post evaluation, having significant effects and lower worse responses in improving frailty. In addition, it can improve functional capacity, balance, and ability in the basic activities of daily living.

## Figures and Tables

**Table 1:** Patients’ baseline demographic and clinical characteristics.

*N*		101
Age		73.09 ± 12.62
Height (cm)		164.73 ± 17.39
Weight (kg)		70.51 ± 14.11
Body Mass Index		25.44 ± 4.05
Days in intensive care unit		19.74 ± 29.42
Previous falls *		0.37 ± 0.82
Previous falls with admission *		0.22 ± 0.56
Fall risk with previous falls	Without risk	32 (31.7%)
	With risk	69 (68.3%)
Fall risk with previous falls with admission	Without risk	79 (78.2%)
	With risk	22 (21.8%)
Sex	Male	56 (55.4%)
	Female	45 (44.6%)
Mild dementia	No	101 (100.0%)
Dominance	Right	101 (100.0%)
Central Sensitization Inventory B score (CSIB) **		
Restless legs	No	100 (99.0%)
	Yes	1 (1.0%)
Chronic fatigue	No	97 (96.0%)
	Yes	4 (4.0%)
Fibromyalgia	No	99 (98.0%)
	Yes	1 (1.0%)
Disorders in the temporomandibular joint	No	100 (99.0%)
	Yes	1 (1.0%)
Irritable colon	No	99 (98.0%)
	Yes	2 (2.0%)
Chemical sensitivity ***	No	100 (99.0%)
	Yes	1 (1.0%)
Cervical injury	No	100 (99.0%)
	Yes	1 (1.0%)
Diagnostics	Cure for pressure ulcer in the sacrum	18 (17.8%)
	Physical deterioration after pneumonia	95 (94%)
	Hip fracture	9 (8.9%)
	Stroke	5 (5%)
	Critical patient polyneuropathy	17 (16.8%)
Clinical history	Ischemic heart disease	13 (12.9%)
	Diabetes mellitus	34 (33.7%)
	Dyslipidemia	36 (35.6%)
	Smoker	15 (14.9%)
	Arterial hypertension	62 (61.4%)
	Heart failure	14 (13.9%)
	Obesity	19 (18.8%)

* Previous falls: previous falls in the past year; previous falls with admission: previous falls in the past year with hospital admission. ** Refers to previous history, not to the current COVID-19 episode *** Multiple chemical sensitivity: It is an acquired, chronic disorder characterized by the appearance of recurrent symptoms in response to exposure to chemical compounds at concentrations that are not considered toxic to the general population. It is usually related to the following symptoms: difficulty breathing, fatigue, dizziness, allergies, itching, nausea, fainting and cardiovascular alterations. Data expressed with mean ± standard deviation or with absolute and relative values (%).

**Table 2:** Quantitative outcome variables.

	Pretreatment	Postreatment	Average Difference (95%CI)	*p* Value ^a^	r (95%CI)
*n*	101	101			
Barthel Index score	31.29 ± 23.47	63.86 ± 28.63	33.333 (25.326, 41.257)	<0.001	0.859 (0.838, 0.869)
Romberg’s test (s)	18.53 ± 15.17	27.38 ± 18.20	9.853 (7.456, 13.21)	<0.001	0.754 (0.659, 0.819)
Semitandem test (s)	18.79 ± 13.71	20.49 ± 14.71	6.161 (4.162, 8.332)	<0.001	0.563 (0.456, 0.646)
Tandem test (s)	13.14 ± 10.37	14.43 ± 9.93	6.605 (4.485, 9.804)	<0.001	0.416 (0.26, 0.508)
Chair Stand test (s)	24.47 ± 13.25	17.38 ± 10.88	−7.016 (−11.39, −3.248)	<0.001	0.535 (0.451, 0.614)
4-Meter Walk Gait Speed test (s)	16.04 ± 9.65	11.60 ± 9.42	−3.834 (−7.062, −0.964)	<0.001	0.673 (0.597, 0.734)
Timed Up and Go test (s)	23.43 ± 12.56	16.44 ± 12.23	−8.024 (−12.026, −4.683)	<0.001	0.701 (0.642, 0.757)
Medical Research Council Sum Score	44.26 ± 12.34	54.99 ± 6.53	9.625 (7.333, 12.096)	<0.001	0.791 (0.705, 0.854)
Handgrip strength (kg)	8.21 ± 5.53	11.79 ± 5.90	3.772 (2.783, 4.671)	<0.001	0.847 (0.803, 0.868)
Trunk Control Test score	65.87 ± 26.17	87.16 ± 19.15	18.75 (12.099, 25.201)	<0.001	0.754 (0.655, 0.83)
Tineti Balance score	7.21 ± 4.33	12.08 ± 3.07	2.875 (1.631, 4.512)	<0.001	0.856 (0.837, 0.868)
Tinetti Gait score	6.36 ± 3.05	9.77 ± 2.07	2.583 (1.631, 3.626)	<0.001	0.853 (0.823, 0.87)
Tinetti-POMA total score	13.56 ± 7.01	21.85 ± 4.74	5.458 (3.5, 7.851)	<0.001	0.862 (0.849, 0.868)
Functional Gait Index score	2.73 ± 0.96	4.63 ± 1.45	2.25 (1.833, 2.679)	<0.001	0.873 (0.864, 0.88)
Short Physical Performance Battery score	3.08 ± 3.07	6.59 ± 3.42	2.583 (1.607, 3.292)	<0.001	0.865 (0.854, 0.872)

Data expressed with mean ± standard deviation or with absolute and relative values (%). 95%CI: 95% confidence interval. r: non parametric effect size. ^a^ significative if *p* < 0.05.

**Table 3:** Qualitative outcome variables.

		Pretreatment	Postreatment	*p* Value ^a^	Cohen’s G
*n*		101	101		
Categorized Romberg’s test (s)	<10 s	47 (46.5%)	14 (13.9%)	<0.001	0.5
	>10 s	54 (53.5%)	87 (86.1%)		
Categorized semitandem test (s)	<10 s	65 (64.4%)	38 (37.6%)	<0.001	0.5
	>10 s	36 (35.6%)	63 (62.4%)		
Categorized tandem test (s)	<3 s	72 (71.3%)	31 (30.7%)	<0.001	0.5
	3.1–9 s	11 (10.9%)	20 (19.8%)		
	>9 s	18 (17.8%)	50 (49.5%)		
Categorized 4-Meter Walk Gait Speed test (s)	Unable	57 (56.4%)	24 (23.8%)	<0.001	0.457
	<8.71 s	29 (28.7%)	29 (28.7%)		
	6.20–8.7 s	5 (5.0%)	7 (6.9%)		
	4.83–6.20 s	4 (4.0%)	17 (16.8%)		
	<4.82 s	6 (5.9%)	24 (23.8%)		
Categorized Chair Stand test (s)	Unable	32 (31.7%)	0 (0.0%)	<0.001	0.487
	>16.7 s	48 (47.5%)	38 (37.6%)		
	13.70–16.69 s	14 (13.9%)	29 (28.7%)		
	11.20–13.69 s	5 (5.0%)	26 (25.7%)		
	<11.19 s	2 (2.0%)	8 (7.9%)		
Categorized Timed Up and Go test (s)	Without risk	83 (82.2%)	84 (83.2%)	1	0.5
	With risk	18 (17.8%)	17 (16.8%)		
Vivifrail fragility level	A	5 (5.0%)	4 (4.0%)	<0.001	0.5
	A+	58 (57.4%)	21 (20.8%)		
	B	8 (7.9%)	14 (13.9%)		
	B+	15 (14.9%)	10 (9.9%)		
	C	9 (8.9%)	22 (21.8%)		
	C+	2 (2.0%)	4 (4.0%)		
	D	4 (4.0%)	22 (21.8%)		
	D+	0 (0.0%)	4 (4.0%)		
Categorized Barthel Index score	Independent (100 points)	0 (0.0%)	11 (10.9%)		
	Slight dependency (91–99 points)	0 (0.0%)	10 (9.9%)	<0.001	0.5
	Moderate dependency (61–90 points)	12 (11.9%)	35 (34.7%)		
	Severe dependency (21–60 points)	45 (44.6%)	33 (32.7%)		
	Total dependency (0–20 points)	44 (43.6%)	12 (11.9%)		

Data expressed with absolute and relative values (%). ^a^ significative if *p* < 0.05.

**Table 4:** Risk of falls.

		Pretreatment	Postreatment	*p* Value ^a^	Cohen’s G
*n*		101	101		
Tinetti–POMA risk	High risk	76 (75.2%)	27 (26.7%)	<0.001	0.484
	Moderate risk	17 (16.8%)	32 (31.7%)		
	Low risk	8 (7.9%)	42 (41.6%)		
Timed Up and Go test (s) risk	Frail adult (14–24 s)	19 (18.8%)	14 (13.9%)	<0.001	0.484
	Ambulation with technical assistance and dependent on ADLs (>30 s)	49 (48.5%)	16 (15.8%)		
	Post-hip surgery (24–30 s)	12 (11.9%)	7 (6.9%)		
	No risk (<14 s)	21 (20.8%)	64 (63.4%)		
Data expressed with absolute and relative values (%).					
ADLs: Daily Life Activities.					
^a^ significative if *p* < 0.05.					

Data expressed with absolute and relative values (%). ADLs: Daily life activities. ^a^ significative if *p* < 0.05.

## Data Availability

The data presented in this study are available on request from the corresponding authors. The data are not publicly available due to ethical restrictions.
